# Supplementary material for: Detailed mapping of the complex fiber structure and white matter pathways of the chimpanzee brain
Source: Nat Methods. 2024 Jun 3;21(6):1122–30. doi: 10.1038/s41592-024-02270-1 (PMC11166572; doi:10.1038/s41592-024-02270-1)
Supplement: Supplementary file 1 — Supplementary Notes 1–6, Supplementary Figs. 1.1– 6.1 and Supplementary Table. [file 41592_2024_2270_MOESM1_ESM.pdf]

nature methods

Supplementary information

https://doi.org/10.1038/s41592-024-02270-1

Detailed mapping of the complex fiber structure and white matter pathways of the chimpanzee brain

In the format provided by the authors and unedited

1. Tissue quality

1.1. Histological examination

The chimpanzee brain was extracted *postmortem*. The brain remained undamaged from extraction, except for two small cuts in the prefrontal cortex and cerebellum. The necropsy reports noted mild hemostasis in the meninges with no other macroscopically visible pathological findings (Supplementary Fig. 1.1a).

The cellular arrangement and myelin distribution appeared normal and without signs of neuropathological processes or tissue decay (Supplementary Fig. 1.1b i-viii). The resident microglia (Supplementary Fig. 1.1b iii, vii) and astrocytes (Supplementary Fig. 1.1b iv, viii) were regularly distributed without substantial signs of activation. Bergmann glia were distinct and subtly branched through the molecular layer to the cerebellar cortical surface (Supplementary Fig. 1.1b vii, viii). A visual screening for age-related neuropathological changes (Aβ plaques and vessel-associated amyloid angiopathy; hyperphosphorylated or aggregated tau protein in neurofibrillary tangles) was negative. In summary, the results of the cerebellar specimen indicated an excellent condition of the brain sample with well-preserved tissue characteristics and no substantial signs of neuropathology.

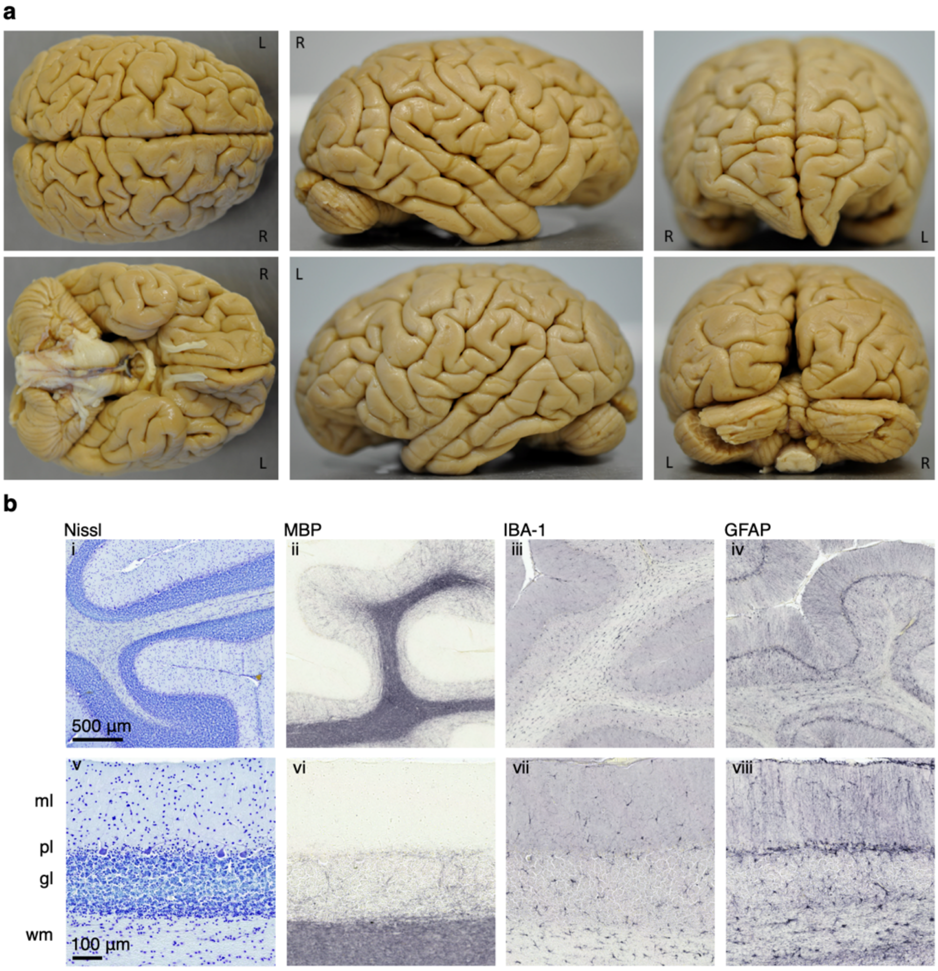

Supplementary Fig. 1.1: (a) Chimpanzee brain after extraction. Photographs of the extracted brain from all sides. **(b)** Brain Histology. Histological assessment of tissue quality in the cerebellum i-iv: Cytoarchitecture (Nissl stain, i) and distribution patterns of myelin (MBP, ii), microglia (IBA-1, iii), and astroglia (anti-GFAP, iv). v-viii: details of cyto- and myeloarchitecture and distribution patterns of microglia and astroglia. Abbreviations: ml = molecular layer, pl = Purkinje cell layer, gl = granular layer, wm = white matter.

1.2. Electron microscopy ultrastructure

Electron microscopic evaluation of the brain tissue ultrastructure confirmed the relatively high tissue quality observed in classical histology (Supplementary Fig. 1.1b; Supplementary Fig. 1.2b-d). Only partially frayed myelin sheaths, shrunken axons, and some tissue voids were observed (Supplementary Fig. 1.2b-d). This degree of ultrastructural tissue damage is acceptable given the initial fixation of the brain in 4% paraformaldehyde after a four-hour *postmortem* interval. Based on the assessment of the tissue ultrastructure and the overall integrity of the myelin sheaths, we conclude that the tissue quality is sufficient for high-quality *postmortem* reconstruction of fiber tracts. We would like to highlight the possibility that the fixative may penetrate the cerebellum before other brain regions, thereby potentially resulting in higher quality tissue evaluations. To provide an indication of the remaining tissue quality, we performed an dMRI analysis of a highly anisotropic white matter brain structure.

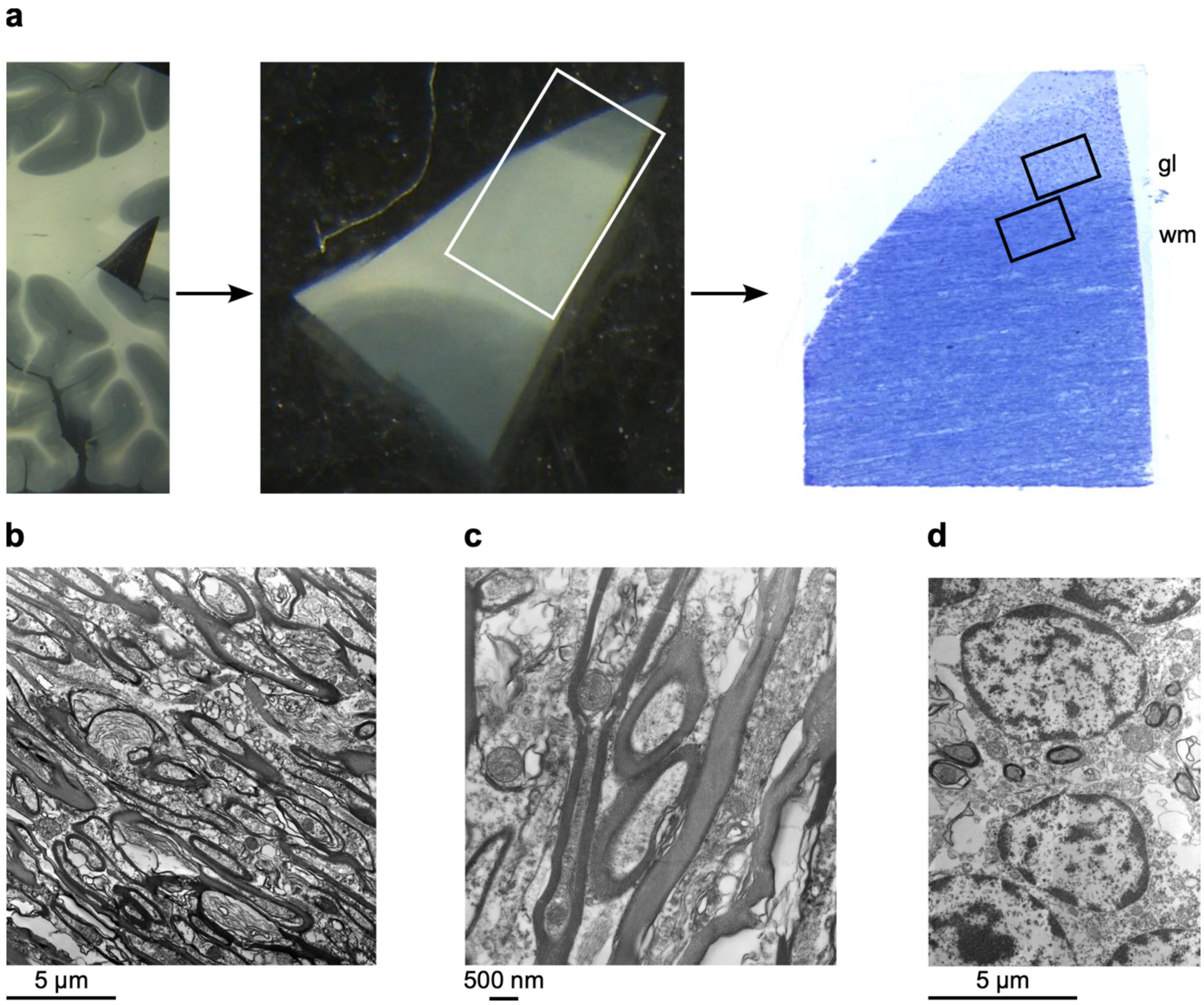

Supplementary Fig. 1.2: (a) Electron microscopy regions. Photomicrographs of a 70µm vibratome section (upper left) showing the region of interest (ROI) extracted for electron microscopical embedding and examination (upper right) and the corresponding toluidine blue-stained semithin section (0.5µm) (lower middle). The ROIs used for electron microscopy are indicated. **(b)** Electron micrograph of white matter ROI. Scale bar 5µm. **(c)** Electron micrograph of white matter ROI with higher magnification. Scale bar 500nm **(d)** Electron micrograph of the granular layer (GL) ROI. Scale bar 5µm

1.3. Diffusion anisotropy

To further assess the tissue quality of the sample, the fractional anisotropy (FA) of the corpus callosum (cc) was compared against chimpanzee *in vivo* values. For *postmortem* samples of high tissue quality, FA values within the cc should be comparable or even higher than *in vivo* (due to reduced partial volume effects at high resolution)[1].

To evaluate this, the FA of the sample was extracted from a region within the cc (Supplementary Fig. 1.3). The mean FA in the *in vivo* chimpanzee cc was obtained from the literature.[2] The here presented *postmortem* sample had an FA of 0.62 ± 0.08 (mean ± sd) inside the cc. The *in vivo* FA of the chimpanzee cc from literature was 0.49 ± 0.06 (mean ± sd). The high DTI values for fractional anisotropy within a homogeneous fiber region are an indication of low tissue degradation and a high tissue quality of the sample.

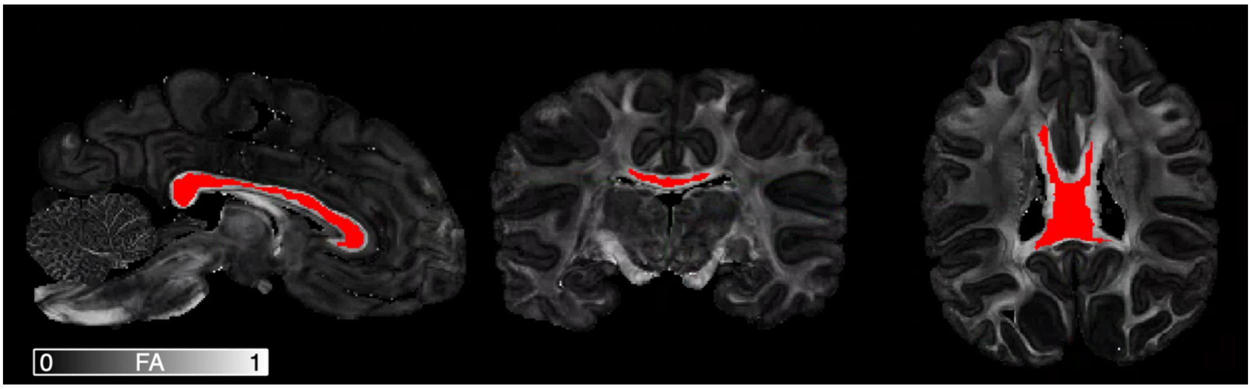

Supplementary Fig. 1.3: The FA map of the sample overlaid with the cc region used for the *in vivo* comparison (displayed in red).

2. Data quality

2.1. Signal-to-noise ratio

We computed a voxel-wise signal-to-noise ratio (SNR) map as the ratio of the first b=0 volume and the standard deviation σ extracted from the acquired noise map (Supplementary Fig. 2.1). Depending on the brain region and tissue type, the SNR ranges up to SNR=300 (mean). The SNR of the data over the whole brain is SNR=83.6 (mean). The SNR of the WM is about SNR=70 (mean).

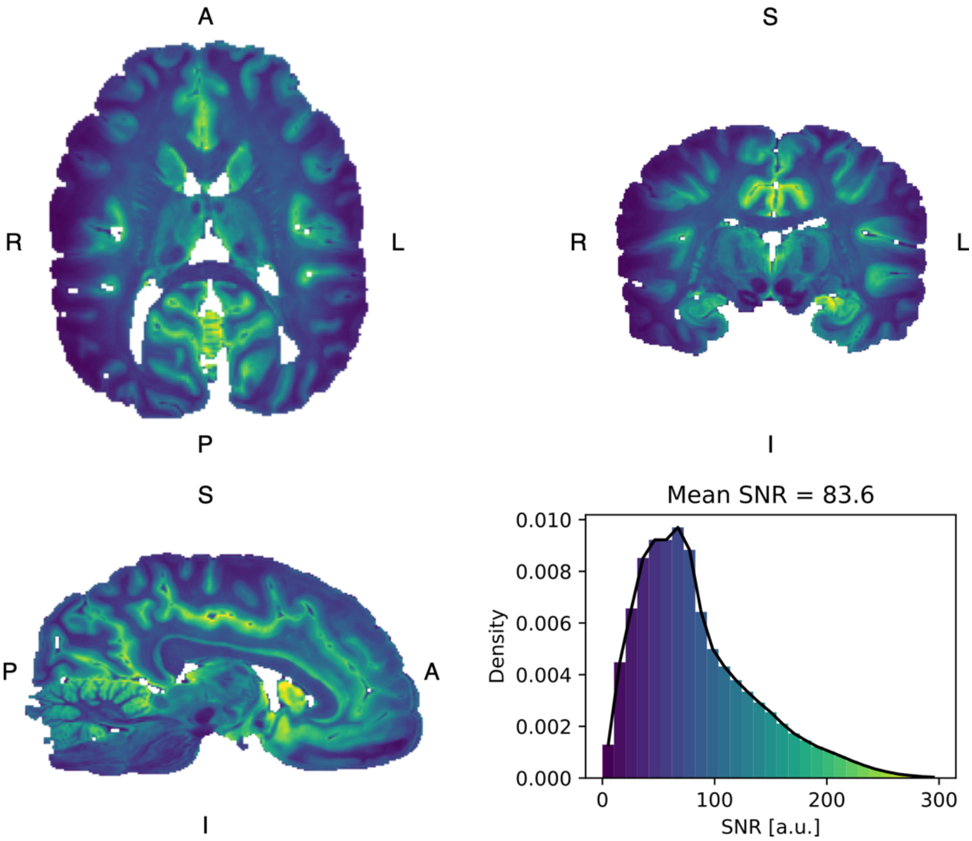

Supplementary Fig. 2.1: Whole-brain signal-to-noise ratio (SNR) map along with the color-coded histogram.

2.2. Test-retest stability assessment

Postmortem dMRI is one of the most challenging acquisition modalities for MRI hardware, as it leverages heavy use gradients over an extended time, to achieve a high image quality. The strong electrical currents required for this task may heat system components during the acquisition process, potentially leading to measurement instabilities. Therefore, the reproducibility of our dMRI measurements and local tissue modeling was assessed in the context of a test-retest evaluation. The assessment focused on the stability of the dMRI signal, DTI FA, LSD kernel ratio, as well as fiber orientation estimations.

Test-retest reproducibility proved excellent across all modalities, as indicated by very low coefficients of variation (CoV_{signal}=0.02, CoV_{FA}=0.05, CoV_{ratio}=0.04). Bland Altman plots illustrate high agreement and low bias between test and retest (Supplementary Fig. 2.2a-c). The reproducibility of fiber orientation estimations was very high: Using DTI, the peak of the angle distribution between the main fiber orientations from the test and retest acquisitions in WM was 5.4° (Supplementary Fig. 2.2d). LSD resulted in a distribution peak of 5.4° for primary and 6.0° for secondary fiber orientations, respectively (Supplementary Fig. 2.2e). Thus, LSD

allowed robust mapping of multiple fiber orientations within a single voxel, while retaining a high level of reproducibility, similar to the far more simplistic DTI model.

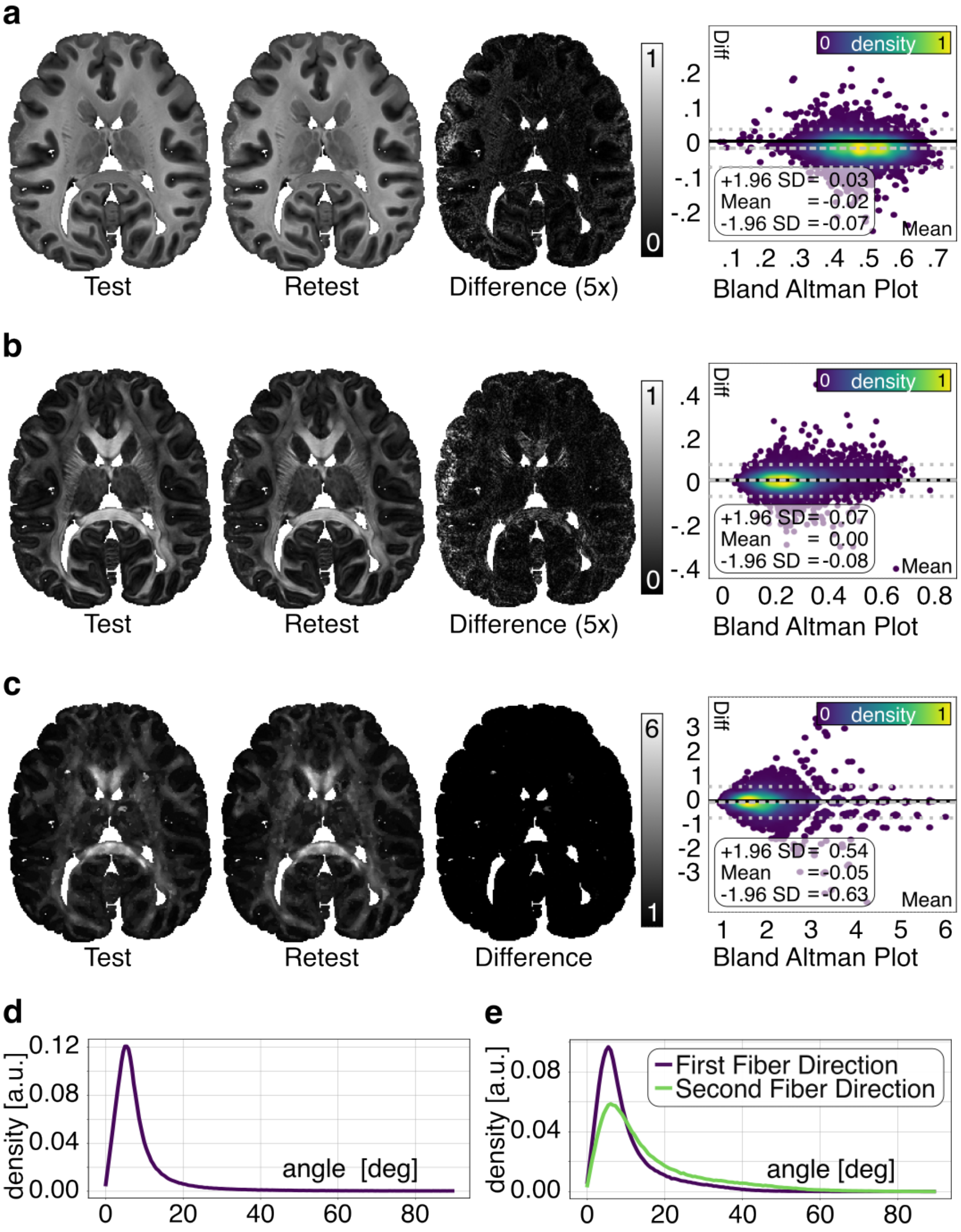

Supplementary Fig. 2.2: (a) Normalized mean dMRI Signal. Test-retest analysis for normalized mean dMRI signal. Figure displays test data, retest data, difference data, and a Bland Altman Plot. **(b)** DTI Fractional Anisotropy. Test-retest analysis for DTI FA data. Figure displays test data, retest data, difference data, and a Bland Altman Plot. **(c)** LSD deconvolution ratio. Test-retest analysis for LSD deconvolution ratios. Figure displays test data, retest data, difference data, and a Bland Altman Plot. **(d)** DTI fiber direction deviation. Distribution of difference in fiber orientation between test and retest data in WM using DTI. **(e)** LSD fiber direction deviation. Distribution of differences in fiber orientation between test and retest data in two fiber regions of WM using LSD.

3. Success factors for high-quality MRI acquisition

The ability to acquire such high-resolution dMRI and MR-microscopy data was due to a combination of three main factors:

(i) *Postmortem* Measurement:

The *postmortem* approach allowed acquiring MRI data continuously over several days (i.e., four days in total). Extended acquisition times can provide a temporal averaging effect that increases the otherwise low signal-to-noise ratio (SNR) of dMRI data.

(ii) Ultra-High-Field MRI System:

A specialized preclinical MRI system with a magnetic field strength of 9.4T, generated a strong MR signal. Further, only through the deployment of an MRI system with a 30 cm bore width, we were able to fit an entire chimpanzee brain into the scanner. Moreover, the acquisition benefited from strong imaging gradients of G = 300 mT/m, vital to generate sufficiently strong diffusion weighting in *postmortem* scans.

(iii) Specialized Diffusion MRI (dMRI) Sequence:

To optimize the signal-to-noise ratio (SNR) of the acquisition, we employed a dedicated sequence, designed for *postmortem* dMRI. Specifically, we leveraged 3D Echo Planar Imaging (EPI) encoding, to generate a higher SNR than typically employed 2D EPI. Further, to increase the signal and reduce distortions (e.g., due to air bubbles) we employed a segmented EPI readout. As a result of this adapted acquisition scheme, no averaging was required to further increase SNR. We used a double EPI readout to prevent Nyquist artifacts. Adiabatic refocusing ensured a homogeneous signal throughout the *postmortem* brain sample. Finally, a pre-assessment of the brain's diffusion properties revealed the sequence settings with the best diffusion contrast.

3.1. Diffusion MRI pre-scan

Formalin fixation of *postmortem* tissue alters its tissue properties. Therefore, the diffusion coefficients of fixed tissue are reduced by an unknown extent[3]. Consequently, standards and experiential guidance from *in vivo* dMRI data no longer apply for *postmortem* dMRI acquisitions. An optimal diffusion-weighting contrast for the *postmortem* brain sample was determined using a series of diffusion-weighted pre-scans. Data were acquired using a 3T Connectom MRI System (Siemens Healthineers AG, Erlangen, Germany), equipped with a high-performance gradient system allowing up to G_{max} = 300 mT/m and a 32 Ch head coil. We acquired pre-scan dMRI data with an isotropic resolution of 2 mm across 22 diffusion shells, ranging from b = 1.000 s/mm^2 to b = 10.000 s/mm^2. Diffusion-contrast was assessed per shell as the signal difference between the primary diffusion orientation and its perpendicular orientation per voxel, ΔS. For the final measurement, we chose the diffusion weighting with the highest median ΔS across the entire brain volume as optimal diffusion weighting.

The computed diffusion contrast increases with growing b-values until it starts to flatten out at roughly b=5000 s/mm^2. For dMRI acquisitions, b=5000 s/mm^2 was chosen to provide an optimal contrast across the whole brain (Supplementary Fig. 3.1).

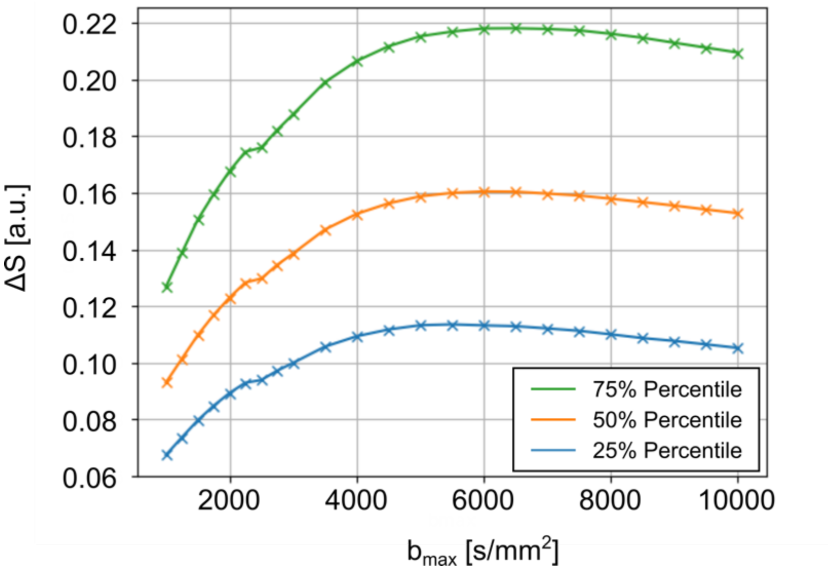

Supplementary Fig. 3.1: Optimal *postmortem* diffusion weighting. Whole-brain diffusion contrast, ΔS, assessed as the difference between signal perpendicular and parallel to the main DTI fiber orientation.

4. Comparison of the chimpanzee resource with existing data

We performed an extensive comparison of the here presented resource atlas with an existing *postmortem* data on chimpanzee brain connectivity from Roumazeilles et al.: "Longitudinal connections and the organization of the temporal cortex in macaques, great apes, and humans". PLoS Biol. (2020)[4]. For brevity, in the following, we refer to datasets by the name of the first author of each respective study (i.e., *Roumazeilles*, *Eichner*).

The *postmortem* dataset from Roumazeilles provides the Ball-and-Stick (BAS) dMRI model[50] reconstruction of a single chimpanzee brain. A direct benchmarking of the *postmortem* data quality is challenging, as the underlying dMRI data is not part of the provided dataset. For an assessment and comparison with the Eichner resource, we chose to evaluate the following parameters.

(i) Contrast-to-Noise Ratio (CNR)

We estimated the dMRI CNR based on the mean primary fiber population (f1) maps of the BAS model, using a kernel-based approach. To that end, we employed FSL bedpostX to fit the BAS model to the Eichner dataset. Visual comparison with the Roumazeilles data indicated a higher CNR in the Eichner resource data (Supplementary Fig. 4.1a, left). We tested this observation quantitatively using a kernel-based approach. For this, we separately estimated the signal contrast and variance from the f1 maps. The f1 contrast in each voxel was estimated through median filtering using a 5×5×5-voxel kernel. As this approach assumes local signal stability, the data masks were eroded to prevent kernels from including both brain and non-brain regions. The local f1 variance was estimated by subtracting the f1 maps from the filtered f1 maps. To reduce the effects of tissue boundaries, the resulting variance map was again median filtered. CNR maps of both datasets were estimated by dividing the filtered f1 maps with the variance map. The resulting CNR maps and distributions (Supplementary Fig. 4.1a, center, right) revealed substantially increased CNR for the Eichner resource (mean CNR = 5.55), compared to the Roumazeilles data (mean CNR = 3.53). As such, the Eichner resource provides a 1.6-fold increase in CNR compared to the Roumazeilles data.

(ii) Capability to Resolve Fiber Crossings

A visual comparison between both datasets indicates a substantially higher number of anatomically meaningful fiber crossings in the Eichner data. The Eichner resource adequately resolves known crossing fiber structures, such as the crossing between cst and cc. (Supplementary Fig. 4.1b, left). For a quantitative evaluation of this observation, we compared the Number of Fiber Orientation (NuFO) maps of the Eichner data with the Roumazeilles data. The Roumazeilles NuFO map was computed by summing the number of provided BAS mean fiber orientations (i.e., dyads, threshold 0.05). For further comparison, we included a CSD NuFO map, computed from a high-quality *in vivo* dataset, randomly selected from the Human Connectome Project (subj. 100307).

The NuFO maps confirm the initial visual observation (Supplementary Fig. 4.1b, right). More than 90% of the Roumazeilles voxels resolve solely one fiber orientation. Less than 10% of the Roumazeilles voxels support a second fiber orientation. For the Eichner data, under 40% of the voxels include only one fiber orientation and more than 60% also include a second fiber orientation. Further, unlike the Roumazeilles data, the Eichner data resolve more than a maximum of two fiber orientations per voxel. As such, the observed NuFO distribution of the Eichner resource resembles the human NuFO distribution from the *in vivo* data (Supplementary Fig. 4.1b, right). Also, the spatial distribution of the Eichner resource NuFOs is closer to the arrangement of fiber crossings in humans (i.e., strong single fiber tracts, such as corpus callosum or optic radiation, many fiber crossings in the deep white matter).

(iii) Image Resolution and Anatomical Detail

The Roumazeilles dMRI data were acquired at an isotropic resolution of 600µm and thus have a 1.7-fold lower resolution than the Eichner resource dMRI data at 500µm. To benchmark the data quality of both datasets, the first fiber orientation (dyad1) of each BAS model output was multiplied by their mean population (mean_f1) to generate a contrast resembling the DTI color-coded FA. This contrast was then visually compared between the two datasets (Supplementary Fig. 4.1c).

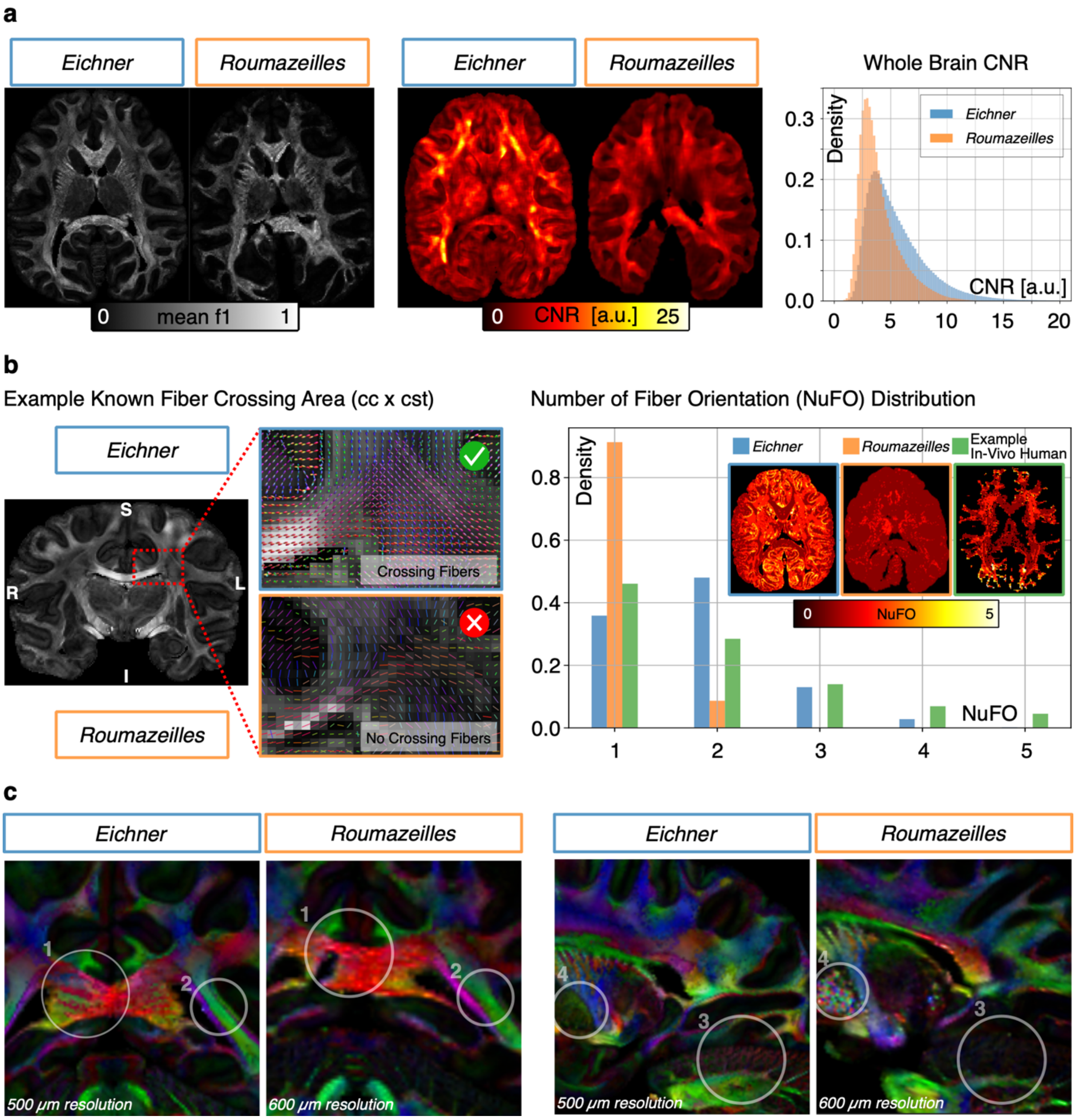

Supplementary Fig. 4.1: Comparison with *postmortem* chimpanzee data from *Roumazeilles et al., 2020.* **(a)** Contrast to noise ratio. Estimation of Contrast to Noise Ratio for Roumazeilles and Eichner data. **(b)** Crossing fiber reconstructions. Left: visual comparison in crossing fiber region (cc, cst crossing) Right: quantitative comparison of fiber NuFO between both *postmortem* resources and *in vivo* human data. **(c)** Image resolution. Comparison of visual data quality between both resources.

In direct comparison between both datasets, it becomes evident that the Eichner resource resolves finer neuroanatomical details due to its higher resolution. For example, the layered structure of the corpus callosum can be seen at the 500µm resolution (Supplementary Fig. 4.1c, 1). Similarly, the elevated resolution allows to recognize very fine fiber structures, such as the boundary between the sagittal stratum and the vertical occipital fasciculus (Supplementary Fig. 4.1c, 2). Similarly, the subtle cerebellar foliate structure is only recognizable at the 500µm resolution of the Eichner resource (Supplementary Fig. 4.1c, 3).

In addition to fine neuroanatomical details, the high tissue quality and optimized imaging strategy allow examining the subcortical gray matter. For instance, the globus pallidus including its internal structure is well visible only in the Eichner resource (Supplementary Fig. 4.1c, 4).

In summary, although the Roumazeilles data provide higher resolution (600 µm isotropic) than the Bryant *in vivo* measurements (1.8 mm isotropic), the data quality exhibits substantial limitations compared to the Eichner resource. The Eichner resource dataset features a considerably higher CNR and allows to adequately reconstruct complex fiber architecture. In addition, the 1.7-fold higher resolution of the Eichner resource allows us to identify even finer neuroanatomical details.

e

5. Assessment and validation of LSD

Impact of acquired diffusion directions on LSD reconstruction

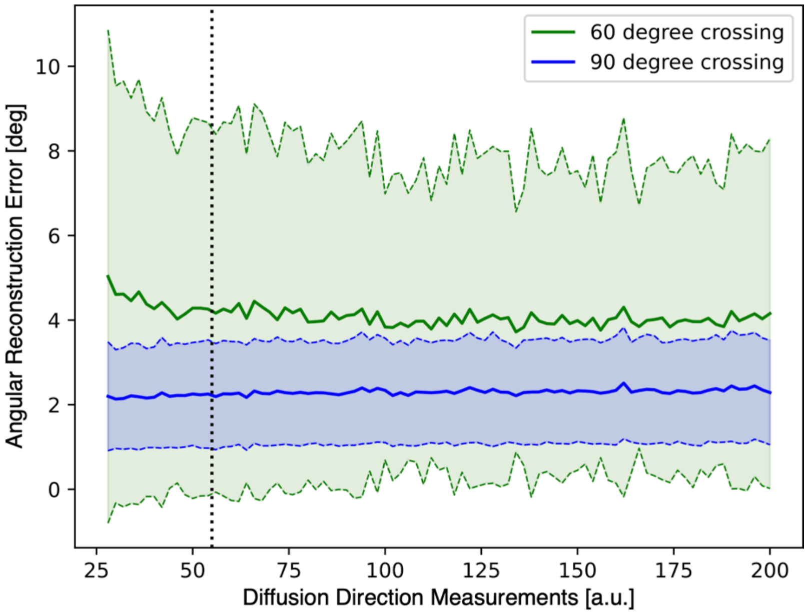

Supplementary Fig. 5.1.1 Angular error of LSD reconstructions for two different fiber configurations as a function of the acquired number of diffusion directions. The solid line indicates the mean error. The dotted line indicates the standard deviation of error.

The data indicate that the reconstruction is insensitive to the number of b-vector directions for the simulated tissue and the diffusion parameters for the 90-degree crossings (Supplementary Fig. 5.1.1). For the 60-degree crossings, we observed a slightly higher average error and larger standard deviation at the very low b-vector counts, which quickly plateaus around N=50 (Supplementary Fig. 5.1.1). We note that the chosen fODF concentration parameter and diffusion kernel ratio were chosen to be slightly on the sharper side of the potential signal, which is expected to be harder to sample properly at a low number of directions. Given this choice of simulated geometry and the observed plateau, we contend that the N=55 directions used in this study is a reasonable choice and we don't expect it to result in any loss of reconstruction accuracy.

Reconstruction accuracy of LSD compared to CSD

Simulation experiment 2peaks-manyK:

Supplementary Fig. 5.1.2 shows some examples of the range of fODF geometries and diffusion kernel shapes in the 2peaks-manyK simulation dataset. The first column shows diffusion tensors with different ratios R, ranging from 1.5 to 8.0. The first row shows 2 peaks 75 degrees intersecting fODF of different concentration parameters κ, ranging from 24 to 8. The remaining glyphs are the noiseless CSA ODFs corresponding to each pair of fODF and diffusion kernel. These ODFs are used as a proxy to highlight the range of possible signals. The central diffusion kernel represents the median ratio used to generate the 2peaks-oneK data set. We note that it is possible to obtain almost identical ODFs by combining a diffusion kernel with a high R (thin) and a fODF with a low κ (thick), or vice versa. In the case of single-shell diffusion, this fact demonstrates the impossibility of disentangling the concentration parameter of the fODF from the ratio of the kernel. This observation is the main justification for the LSD approach.

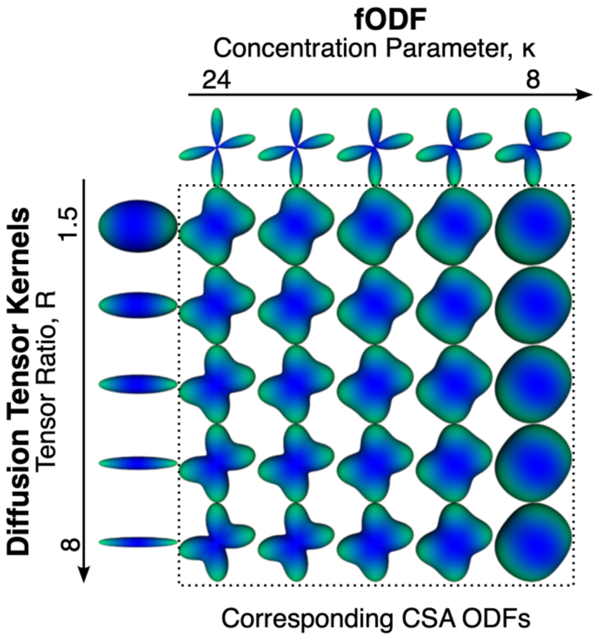

Supplementary Fig. 5.1.2 Different possible ODF geometries for a 75 deg fiber crossing, resulting from different combinations of fODF concentration (i.e., fanning) and diffusion kernels (i.e., microstructural environment).

Supplementary Fig. 5.1.3a summarizes the analysis results of the 2peaks-manyK simulation. Overall, LSD consistently reconstructs about 10% more total cases than CSD and strongly outperforms CSD in the lower crossing angle regime (less than 60 degrees). This is easily explained by the nature of both methods; while CSD deconvolves the data once with a computed kernel, LSD deconvolves the data with 20 different kernels to obtain 20 candidate fODFs. While the underlying algorithm is different, each of these 20 deconvolutions is still somewhat equivalent to CSD, but LSD then performs a model selection over the candidate fODFs. Therefore, it is not surprising that LSD consistently outperforms CSD as long as the underlying model selection is reasonable. The simulations show that LSD substantially outperforms CSD reconstruction over a wide range of crossing angles and SNR values.

Simulation dataset 2peaks-oneK:

Supplementary Fig. 5.1.3b summarizes the analysis results of the 2peaks-oneK simulation. The experiment with the 2peaks-manyK dataset violated the main hypothesis of CSD, the existence of a unique diffusion kernel. Using the 2peaks-oneK dataset, we show that LSD still outperforms CSD when this hypothesis is perfectly satisfied. The 2peaks-oneK dataset is overall easier to reconstruct, as it does not contain challenging voxel geometries such as low diffusivities and/or low ratios. This results in a higher overall STAR metric for both LSD and CSD. However, the difference map shows the same pattern of LSD slightly but consistently outperforming CSD in the high SNR and crossing angle range, with a very large difference below 60 degrees. The simulations show that LSD substantially outperforms CSD reconstruction over a wide range of crossing angles and SNR values, even when the main tissue assumptions of CSD are valid.

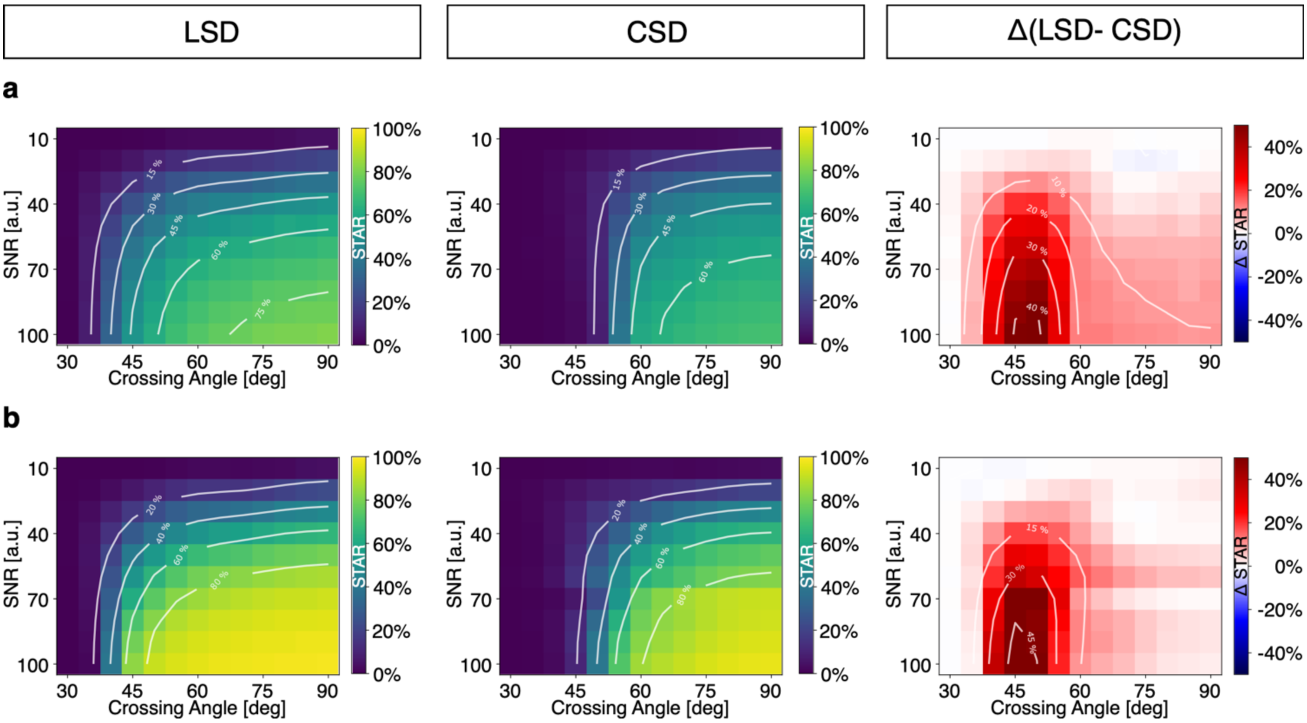

Supplementary Fig. 5.1.3: LSD vs CSD simulation results – proportion of correct fODF reconstructions **(a)** Multiple kernel manyK simulation assuming microstructural heterogeneity. Left: The ratio of successful LSD reconstructions against *in silico* ground truth for various crossing angles and SNR values. Center: The ratio of successful CSD reconstructions against *in silico* ground truth for various crossing angles and SNR values. Right: The percentage point difference between successful LSD and CSD reconstructions for various crossing angles and SNR values. Positive values indicate better LSD reconstruction. **(b)** Single kernel oneK simulation assuming microstructural homogeneity (CSD Assumption). Left: The ratio of successful LSD reconstructions against *in silico* ground truth for various crossing angles and SNR values. Center: The ratio of successful CSD reconstructions against *in silico* ground truth for various crossing angles and SNR values. Right: The percentage point difference between successful LSD and CSD reconstructions for various crossing angles and SNR values. Positive values indicate better LSD reconstruction.

In summary, from the two simulation experiments, we can conclude that LSD reconstructions provide substantially higher accuracy of diffusion reconstruction than the established CSD model over a wide parameter range of fiber configuration and data quality. Due to the large variability of *postmortem* diffusion parameters, we have chosen to perform the simulations using approximately known *in vivo* human diffusion parameters, without limiting the generality of the results. Our simulation results indicate that LSD can also provide better fiber reconstruction in the human *in vivo* context.

5.1. Histological assessment of LSD in human sample

Comparison between LSD and CSD reconstructions

Direct comparison of CSD and LSD reconstructions based on high-resolution data in known human anatomy (Supplementary Fig. 5.2.1) suggests similar effects to those observed in the chimpanzee brain sample. Compared to CSD, LSD fODFs appear sharper and more ordered, confirming the known anatomy of the corona radiata. In contrast, CSD fODFs appear blunt and directionally fragmented. This is particularly evident in the inferior part of the corona radiata (Supplementary Fig. 5.2.1a,b, Magnification 3,4), where the overly sharp CSD kernel estimation resulted in split fODFs, indicating incorrect fiber orientations.

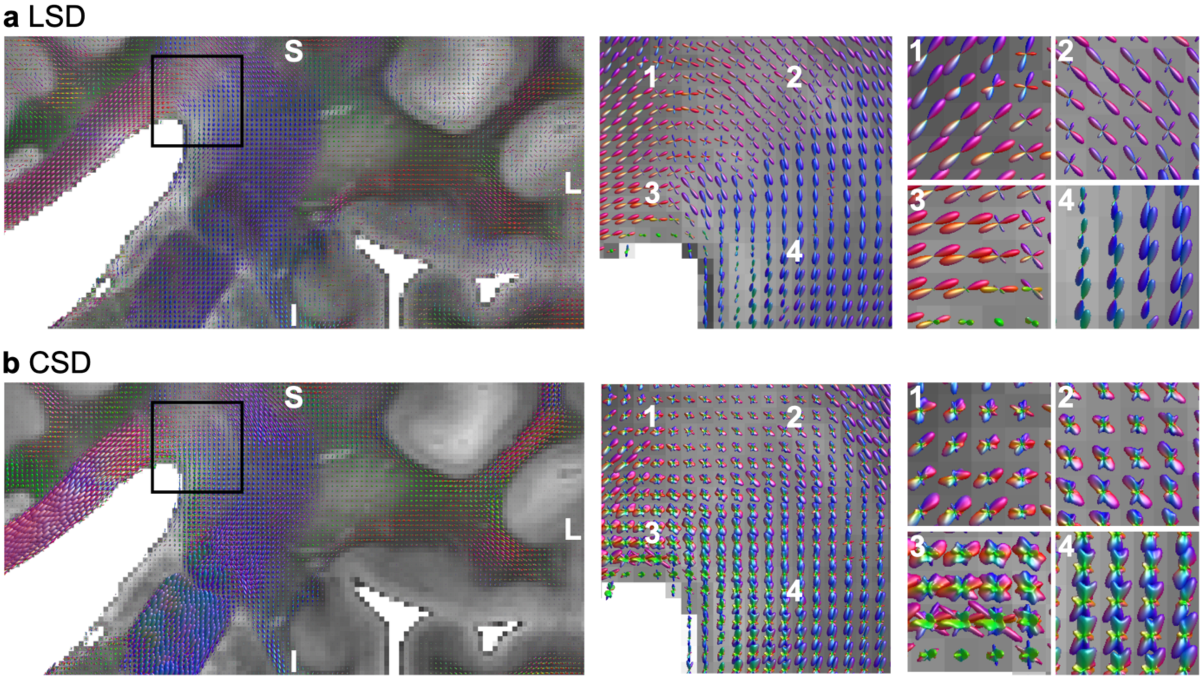

Supplementary Fig. 5.2.1 Comparing LSD and CSD fODF reconstructions in a human *postmortem corona radiata* **(a)** LSD reconstruction alongside four zoomed regions within the corona radiata. The black rectangle indicates the zoomed region. The numbers indicate the further zoomed sub regions. **(b)** CSD reconstruction alongside four zoomed regions within the corona radiata. The black rectangle indicates the zoomed region. The numbers indicate the further zoomed sub regions.

Histological validation of LSD reconstruction

To enable a comparison between LSD 3D fODFs (Supplementary Fig. 5.2.2a) and 2D histology (Supplementary Fig. 5.2.2b), the dMRI fODFs were projected on a 2D space. To do this, the LSD sh coefficients (sh max order 8) of the 3D ODFs were projected onto a fine 3D sphere of 46210 vertices. The deformed sphere was treated as a point cloud and projected onto a 2D plane (coronal surface). The coronal 2D plane was then discretized into 1 deg radial bins (Supplementary Fig. 5.2.2c). We computed 2D ODFs by finding the point with the maximum norm in the plane for each radial bin. Each 2D ODF was min-max normalized and colored according to its mean weighted direction using the HSV color map on the semicircle.

We computed histology-based fiber orientation maps using microscopic autocorrelation[5] (Supplementary Fig. 5.2.2c). For each tile in the histology image, a 2001x2001 crop was selected around the center. This was done to avoid the edges of the tile stitching. For each tile, we then computed the self-correlation using an FFT convolution, normalized by overlap area (100-pixel correlation radius). This central disk was linearly interpolated on a polar grid with radial bins of 0.1 pixels and angular bins of 1 deg. We computed histology ODFs respecting solid angle constraint by multiplying the autocorrelation values by their radius and summing along each radial bin. Each resulting histology ODFs was then min-max normalized and color-coded according to its mean weighted direction using the HSV color map on the semicircle.

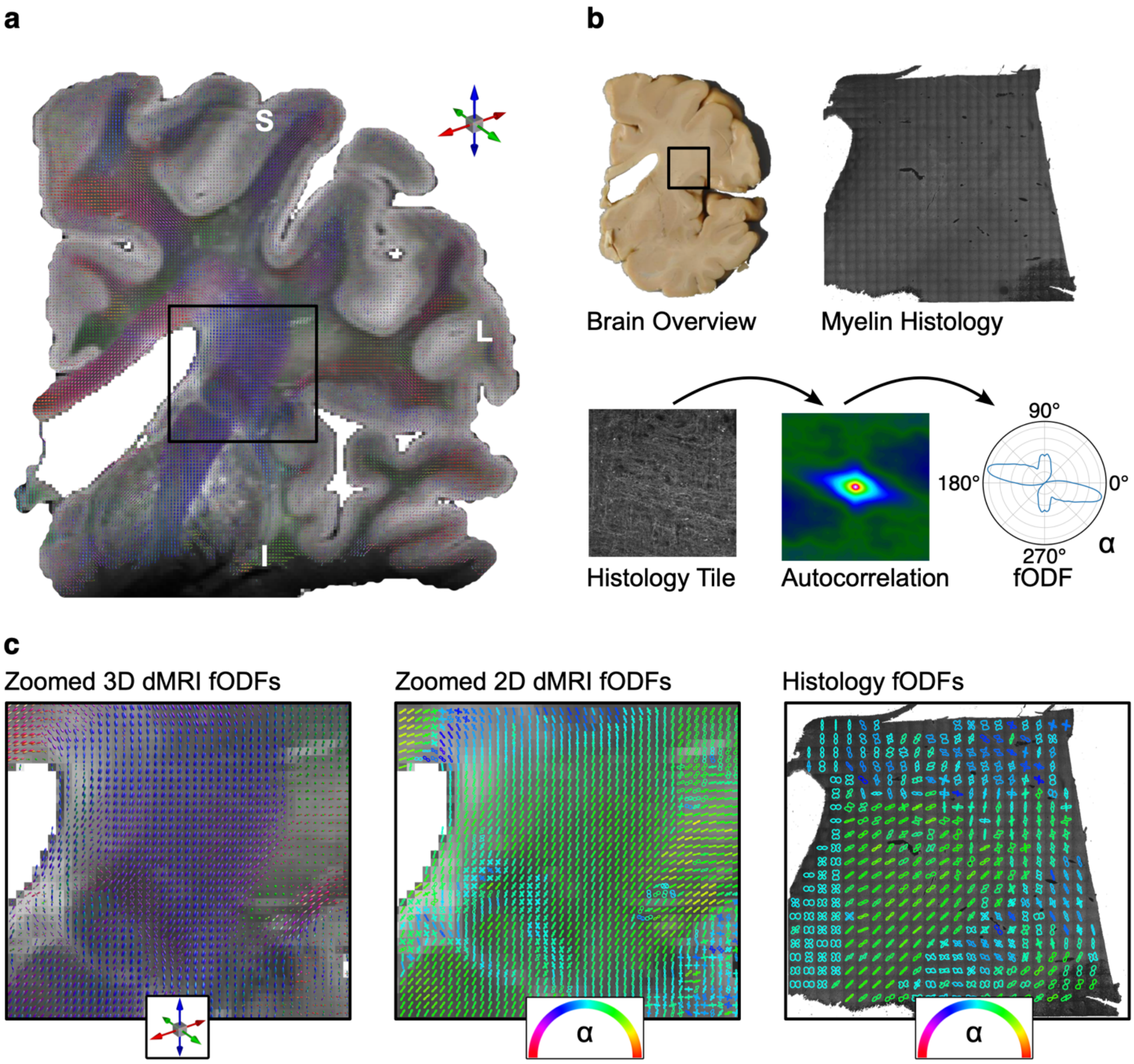

Supplementary Fig. 5.2.2 Comparison of LSD fODF reconstruction and myelin autofluorescence. **(a)** Whole slab LSD reconstruction. LSD fODFs were reconstructed for a 2 cm *postmortem* brain slice (left hemisphere) at 400 µm isotropic resolution. **(b)** Histology fODF reconstruction. Myelin autofluorescence data were acquired for a sample of the *postmortem* slice. 2D histology fODFs were calculated for the histology slice using pixel autocorrelation. **(c)** Comparison between LSD and histology fODFs. The 3D LSD fODFs (left) were converted to 2D by projection onto a coronal plane (middle) and compared with 2D histology fODFs (right). The 2D fODFs are color-coded according to the mean in-plane angle α.

Supplementary Fig. 5.2.2c shows the results of the human corona radiata comparison between LSD reconstruction and myelin histology. The comparison allows us to recognize distinctive features of the fiber structure in the 2D dMRI fODFs as well as in the myelin fiber histology fODFs. For example, the prominent cst (green) and the striatal bridges running transversely to the cst (cyan) are equally visible in both maps. Note that 100% homology between the two images is not expected because (i) both modalities are sensitive to different tissue properties (i.e., water diffusion vs. myelin), and (ii) the histology data are distorted from the tissue sectioning process.

6. Brain data and tractography

6.1. Brain surface and brain sulci

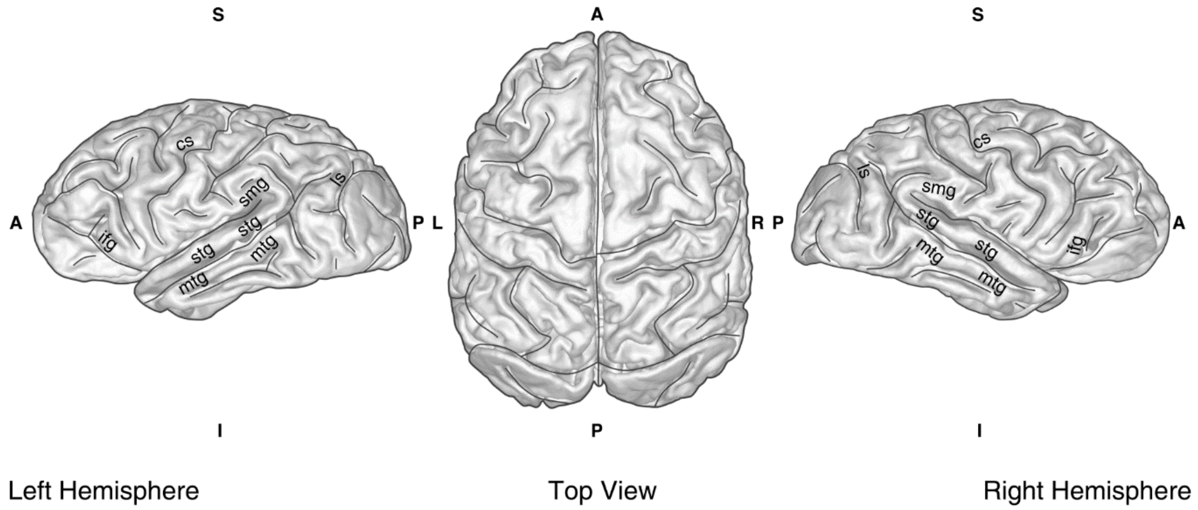

Supplementary Fig. 6.1.1: White matter surface rendering overlaid with drawn brain sulci. Anatomical positions are provided for the central sulcus (cs), inferior frontal gyrus (ifg), lunate sulcus (ls), middle temporal gyrus (mtg), supramarginal gyrus (smg), superior temporal gyrus (stg) in both brain hemispheres.

6.2. White matter fascicle segmentation (alphabetic order)

Arcuate fascicle (af):

The af connects the inferior frontal lobe with the posterior temporal lobe via the parietal lobe. The fascicle was segmented from the full brain tractography by placing selection ROIs in the ventral prefrontal area, the parietal lobe at the level of the supramarginal gyrus, and the temporal lobe. The selected streamlines reached only the most posterior part of the temporal lobe and the most ventral part of the posterior inferior frontal lobe including premotor areas.

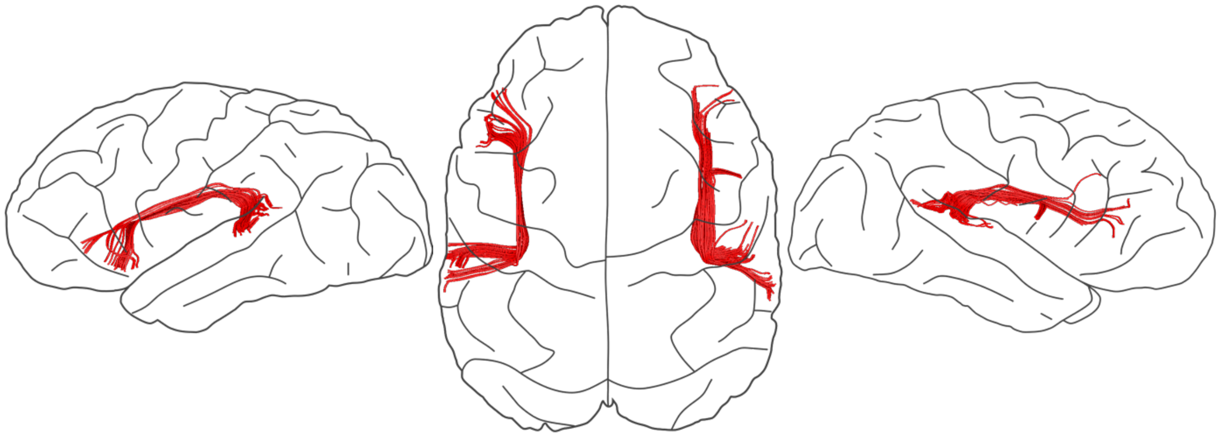

Supplementary Fig. 6.2.1: Arcuate fascicle (af) in both hemispheres. For anatomical orientation, please see Supplementary Fig. 1.1, Supplementary Fig. 6.1.1

Cingulum (cg):

The cg connects medial prefrontal and parietal regions with the parahippocampal gyrus. The segmentation was combined from a dorsal and a ventral component. The dorsal part was segmented by placing selection ROIs in the prefrontal cingulate gyrus covering the cingulate white matter and in the parietal cingulate

gyrus. The ventral part was selected with ROIs in the medial parietal lobe and the ventral parahippocampal gyrus. The selected streamlines connected medial subcallosal, medial frontal, medial parietal, and parahippocampal areas.

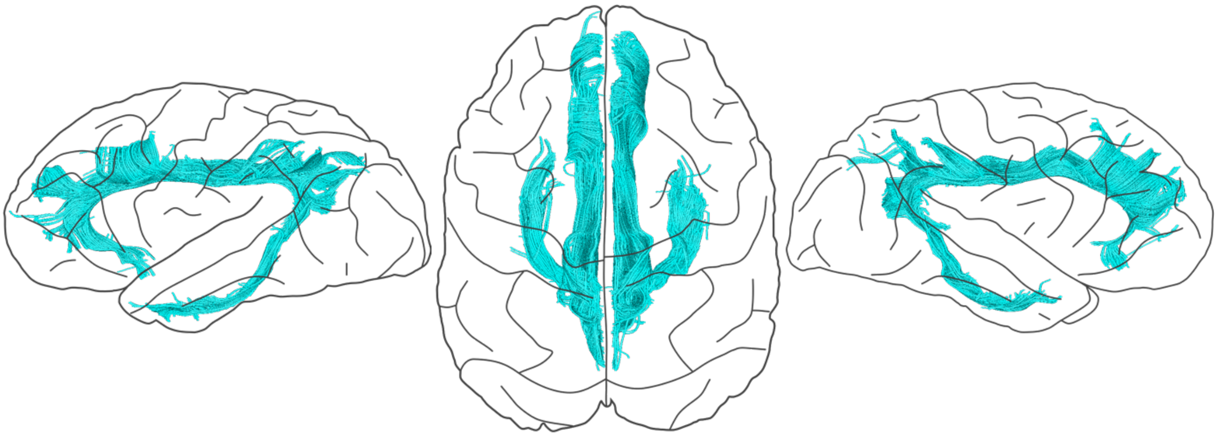

Supplementary Fig. 6.2.2: Cingulum bundle (cg) in both hemispheres. For anatomical orientation, please see Supplementary Fig. 1.1, Supplementary Fig. 6.1.1

Corticospinal tract (cst):

The cst connects primary motor and sensory areas with the spinal cord. It was selected using one ROI covering the pre and postcentral gyrus and one ROI in the spinal cord below the pons. The selected streamlines mainly connected the dorsal parts of the somatomotor system and lateral parts could not be reconstructed with this approach. The pathway is separated into several smaller tracts crossing the brainstem at different positions. One subcomponent crossed the thalamus. Details are visible in the online 3D visualization.

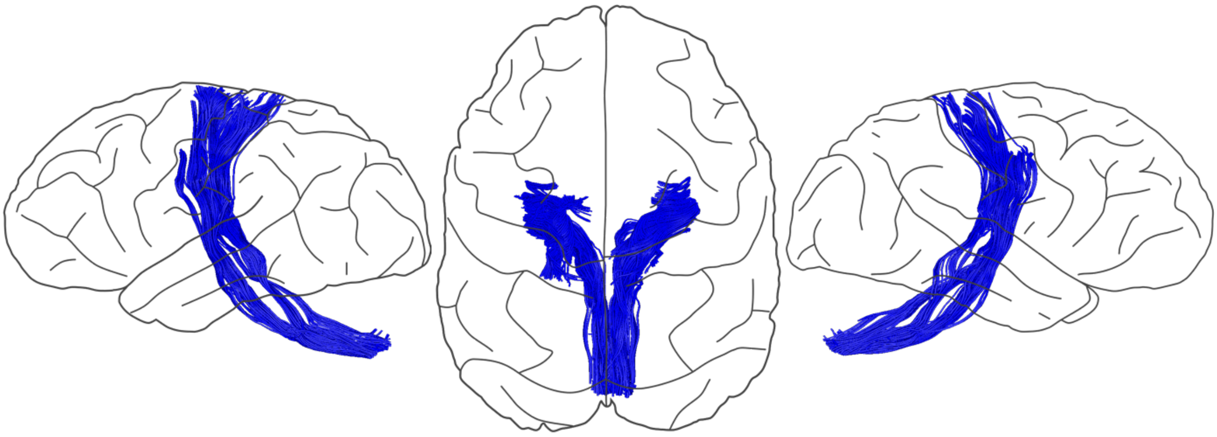

Supplementary Fig. 6.2.3: Corticospinal tract (cst) in both hemispheres. For anatomical orientation, please see Supplementary Fig. 1.1, Supplementary Fig. 6.1.1

Inferior fronto-occipital fascicle (ifof):

The ifof connects prefrontal with temporal and occipital areas. It was selected using one ROI in the ventral prefrontal white matter, a smaller central ROI in the ventral extreme / external capsule, and one ROI in the central portion of the temporal lobe. The segmented pathway mainly connected the occipital lobe with the frontal lobe without dominant temporal projections.

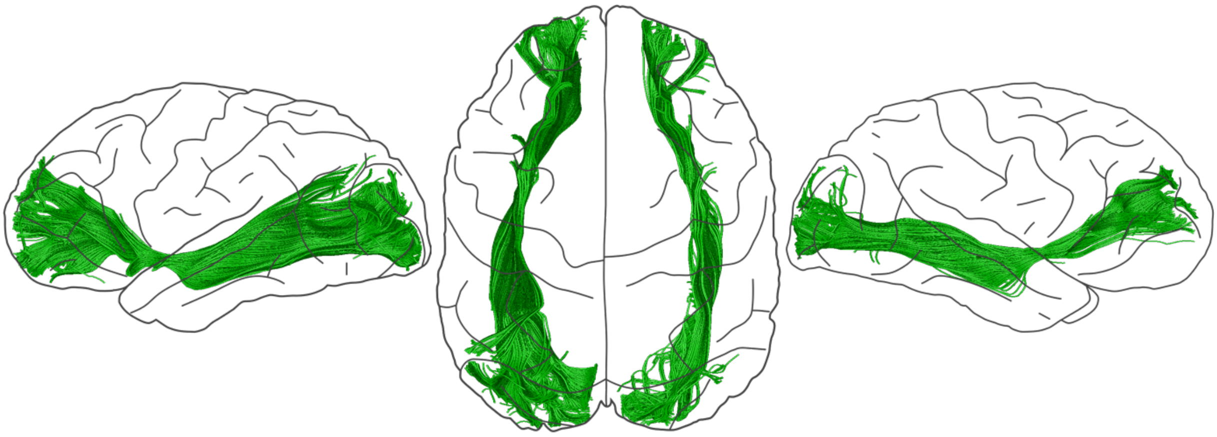

Supplementary Fig. 6.2.4: Inferior fronto-occipital fascicle (ifof) in both hemispheres. For anatomical orientation, please see Supplementary Fig. 1.1, Supplementary Fig. 6.1.1

Inferior longitudinal fascicle (ilf):

The ilf connects different parts of the inferior and middle temporal gyri and the occipital lobe. The selection ROIs were placed in the ventral temporal pole covering the middle and inferior temporal gyrus and in the central portion of the temporal lobe. The pathway connected ventral occipital and posterior temporal regions with the ventral temporal pole.

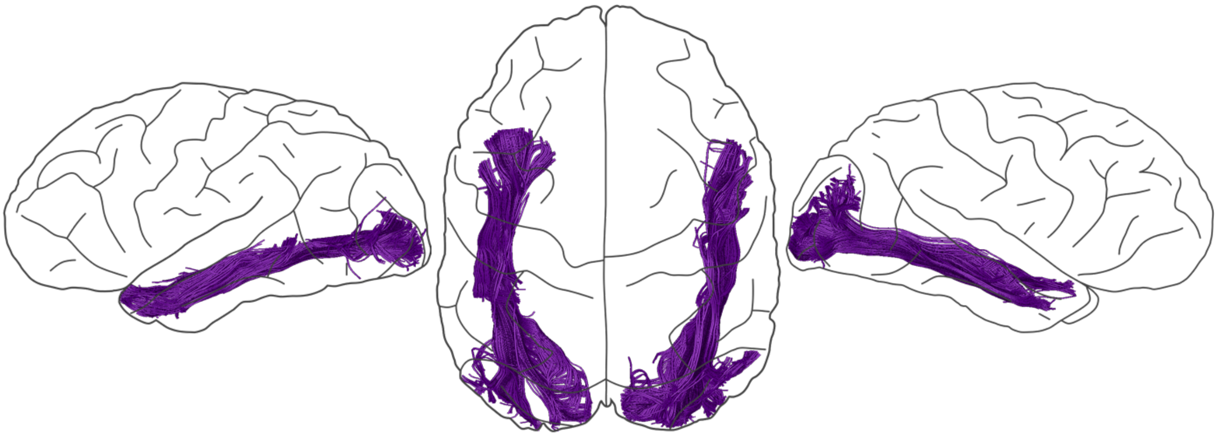

Supplementary Fig. 6.2.5: Inferior longitudinal fascicle (ilf). For anatomical orientation, please see Supplementary Fig. 1.1, Supplementary Fig. 6.1.1

Middle cerebellar peduncle (mcp):

The mcp is an important tract connecting the left and right cerebellum while crossing the cst at the level of the pons. The selection ROIs were placed in the left and the right cerebellum respectively and in the pons. Different crossings of the mcp with the cst could be clearly separated within the pons. Details are visible in the online 3D visualization.

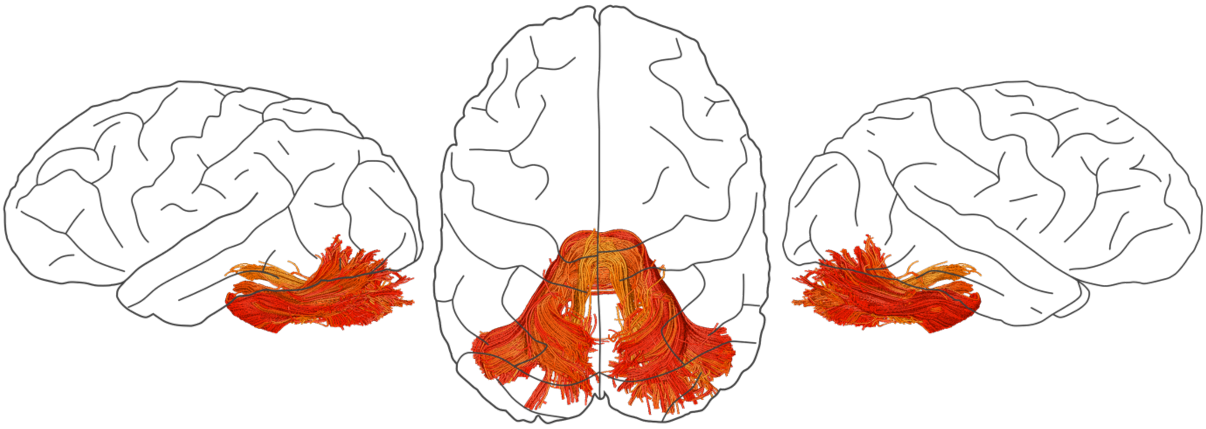

Supplementary Fig. 6.2.6: Middle cerebellar peduncle (mcp). For anatomical orientation, please see Supplementary Fig. 1.1, Supplementary Fig. 6.1.1

Middle longitudinal fascicle (mdlf):

The mdlf is dorsal to the ilf in the white matter of the superior temporal gyrus (stg). It was selected by an ROI in the temporal pole of the stg and an ROI in the central portion of the temporal lobe. It connected the occipital lobe with the superior temporal pole.

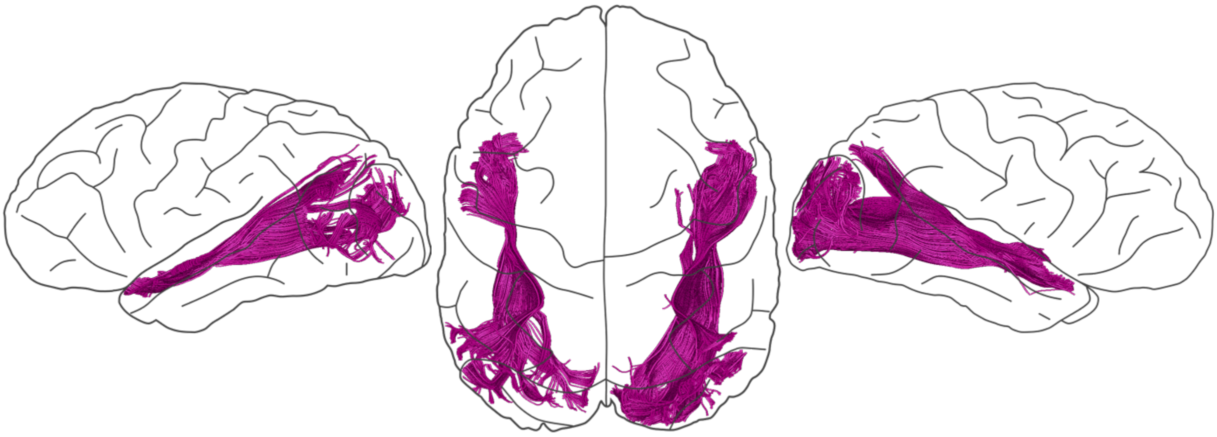

Supplementary Fig. 6.2.7: Middle longitudinal fascicle (mdlf). For anatomical orientation, please see Supplementary Fig. 1.1, Supplementary Fig. 6.1.1

Superior longitudinal fascicle I (slf I):

The slf I connects superior parietal with superior frontal regions. It was selected by placing an ROI in the posterior superior frontal gyrus (sfg) also covering the dorsal part of the precentral gyrus (pcg). A second ROI was placed in the dorsal parietal lobe. The selected pathway connected the dorsal pcg and the posterior sfg to a similar region in the dorsal parietal lobe.

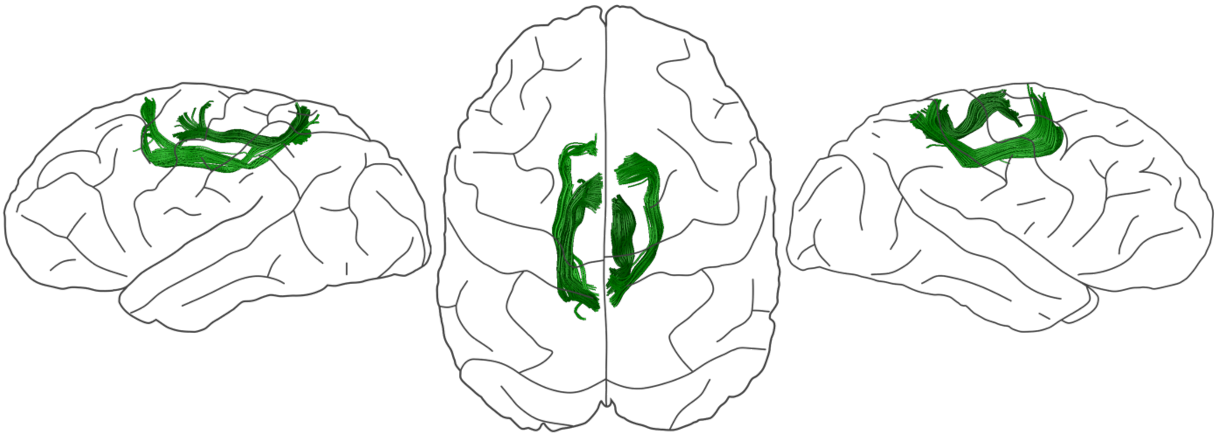

Supplementary Fig. 6.2.8: Superior longitudinal fascicle I (slf I). For anatomical orientation, please Supplementary Fig. 1.1, Supplementary Fig. 6.1.1

Superior longitudinal fascicle II (slf II):

The slf II connects the middle frontal gyrus with the parietal lobe. It was selected by an ROI in the posterior middle frontal gyrus including the corresponding part of the pcg and a second ROI in the parietal lobe. The selected pathway connected the posterior middle frontal gyrus with the anterior superior parietal lobe ventral to the projection of the slf I.

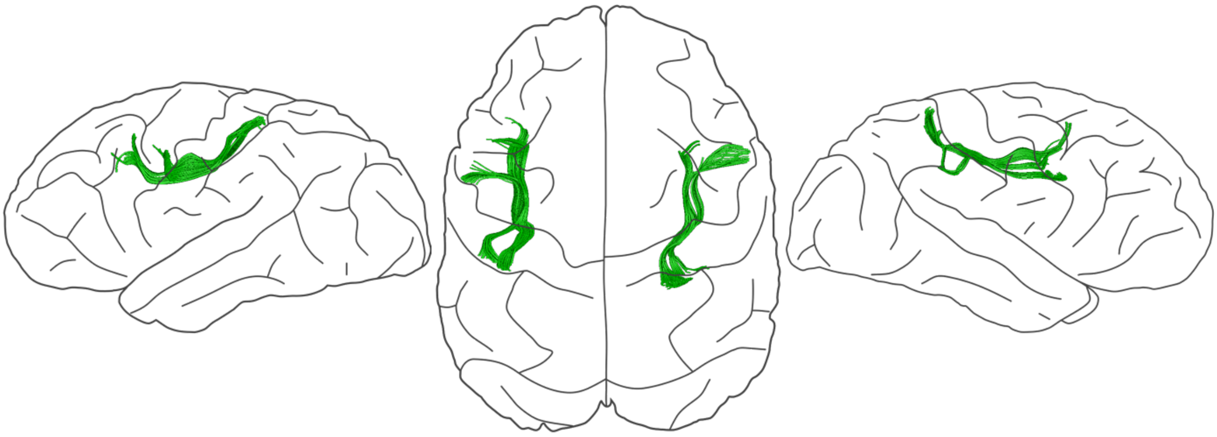

Supplementary Fig. 6.2.9: Superior longitudinal fascicle II (slf II). For anatomical orientation, please see Supplementary Fig. 1.1, Supplementary Fig. 6.1.1

Superior longitudinal fascicle III (slf III):

The slf III connects the inferior frontal gyrus (ifg) and the ventral premotor cortex with the ventral parietal lobe. It was selected by an ROI in the posterior ifg including the ventral pcg and a second ROI in the ventral parietal lobe. The selected pathway connected different parts of the posterior inferior frontal gyrus and the ventral precentral gyrus with the supramarginal gyrus.

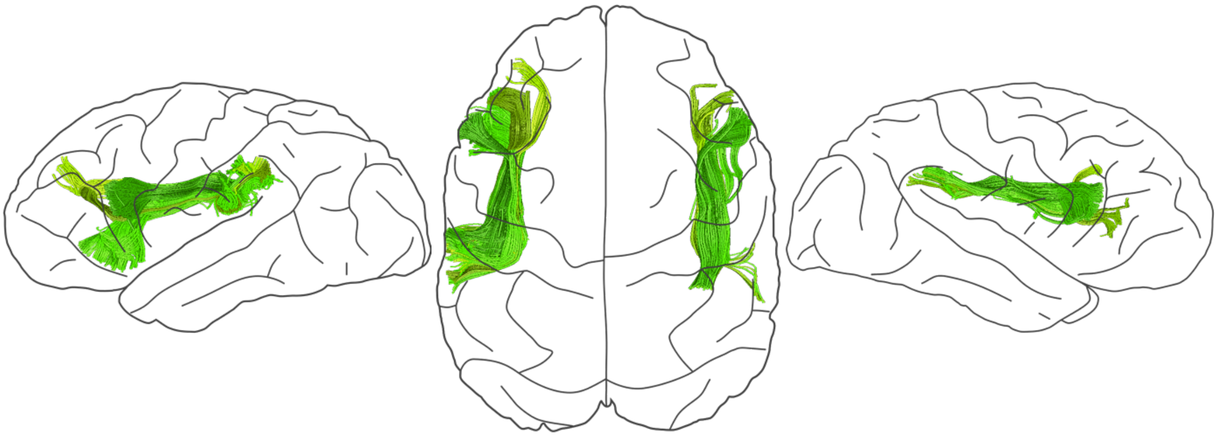

Supplementary Fig. 6.2.10: Superior longitudinal fascicle III (slf III). For anatomical orientation, please see Supplementary Fig. 1.1, Supplementary Fig. 6.1.1

Uncinate fascicle (unc):

The unc connects prefrontal areas with the temporal pole. It was selected by one ROI in the ventral prefrontal frontal lobe anterior to the insula, one ROI in the temporal pole, and a third central ROI in the ventral extreme / external capsule equivalent to the central ROI used for the ifof.

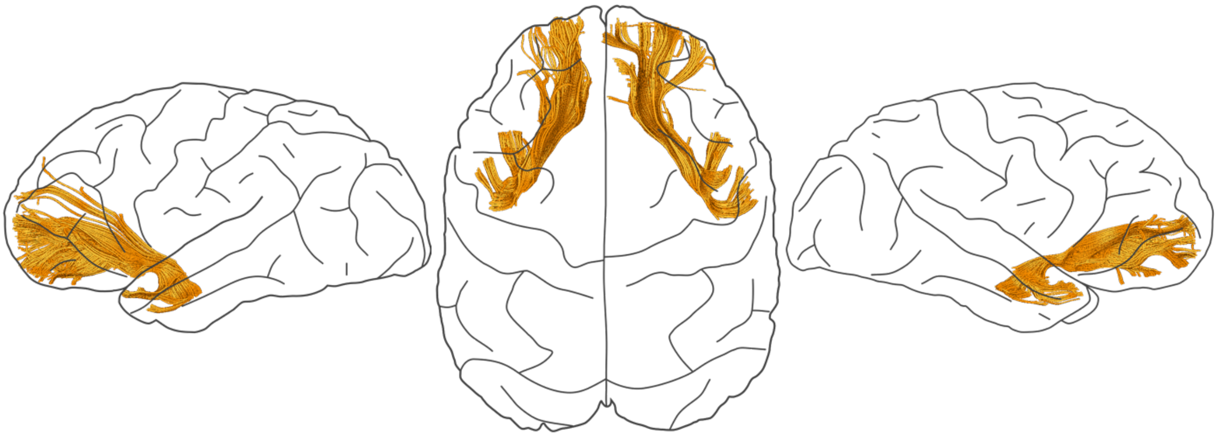

Supplementary Fig. 6.2.11: Uncinate fascicle (unc). For anatomical orientation, please see Supplementary Fig. 1.1, Supplementary Fig. 6.1.1

7. Inter hemispheric connections

The data quality becomes evident when observing inter-hemispheric connections, other than the cc. The size ratios of inter-hemispheric brain connections between the cc and the other commissures have shifted throughout primate evolution. The here observed cross-sectional ratio at the brain midline between the ac and cc for the chimpanzee is 0.022 (marmoset: 0.096, macaque: 0.068, human: 0.015)[6] and between the pc and cc for the chimpanzee it is 0.009 (marmoset: 0.060, macaque: 0.016, human: 0.007)[6]. All ratios are corrected for brain size. This change was potentially driven by evolutionary optimization following the increasing brain size and the associated connectome of higher cognitive frontal and parietal areas[7]. In humans, the cc is the largest and dominant inter-hemispheric connection. However, in monkeys (e.g., macaques or marmosets), other inter-hemispheric connections such as the ac or pc are more prominent than in humans[6]. The strong evolutionary changes of these pathways underline their importance to the evolution of brain connectivity. However, due to limited image resolution of previous chimpanzee dMRI data, the current literature is restricted to a description of only the most prominent inter-hemispheric connections in this species[8] (i.e., cc, ac).

8. Supplementary table – anatomical abbreviations

Structure Name	abbreviation	tissue class
anterior commissure	ac	WM
arcuate fascicle	af	WM
anterior limb of internal capsule	alic	WM
anterior thalamic radiation	atr	WM
caudate nucleus	cau	SCGM
corpus callosum	cc	WM
cerebellar folia	cf	GM
cingulum bundle	cg	WM
corticopontine tract	cpt	WM
corona radiata	cr	WM
corticospinal tract	cst	WM
cerebellar white matter	cwm	WM
fornix	fx	WM
genu of corpus callosum	gcc	WM
external globus pallidus	gpe	SCGM
internal globus pallidus	gpi	SCGM
habenular commissure	hc	WM
inferior colliculus	ico	SCGM
internal capsule	ic	WM
inferior cerebellar peduncle	icp	WM
inferior fronto-occipital tract	ifof	WM
inferior longitudinal fascicle	ilf	WM
lateral geniculate nucleus	lgn	SCGM
lateral lemniscus	ll	WM
lateral ventricle	lv	other
middle commissure	mc	WM
middle cerebellar peduncle	mcp	WM
medial lemniscus	ml	WM
muratoff bundle	mu	WM
optic nerve	on	WM
optic radiation	or	WM
optic chiasm	ox	WM
posterior commissure	pc	WM
pontine crossing tract	pct	WM
posterior limb of internal capsule	plic	WM
pontine nuclei	pn	SCGM
putamen	pt	SCGM
posterior thalamic radiation	ptr	WM
striatal cell bridges	sb	SCGM
splenium of corpus callosum	scc	WM
superior cerebellar peduncle	scp	WM
superior fronto- occipital fascicle	sfo	WM
superior longitudinal fascicle	slf	WM
supramamillar commissure	smc	WM
superior thalamic radiation	str	WM
tapetum	tap	WM
thalamus	th	SCGM
uncinate fascicle	unc	WM
ventral hippocampal commissure	vhc	WM
middle longitudinal fascicle	mdlf	WM

Tissue Classes: Gray Matter (GM), Subcortical Gray Matter (SCGM), White Matter (WM)

9. Supplementary references

1. Sun, S.-W. *et al.* Formalin fixation alters water diffusion coefficient magnitude but not anisotropy in infarcted brain. *Magn. Reson. Med.* **53**, 1447–1451 (2005).
2. Phillips, K. A. & Hopkins, W. D. Topography of the chimpanzee corpus callosum. *PLoS One* **7**, e31941 (2012).
3. Roebroeck, A., Miller, K. L. & Aggarwal, M. Ex vivo diffusion MRI of the human brain: Technical challenges and recent advances. *NMR Biomed.* **32**, e3941 (2019).
4. Roumazeilles, L. *et al.* Longitudinal connections and the organization of the temporal cortex in macaques, great apes, and humans. *PLoS Biol.* **18**, e3000810 (2020).
5. Wedeen, V. J. *et al.* The geometric structure of the brain fiber pathways. *Science* **335**, 1628–1634 (2012).
6. Liu, C. *et al.* A resource for the detailed 3D mapping of white matter pathways in the marmoset brain. *Nat. Neurosci.* **23**, 271–280 (2020).
7. Assaf, Y., Bouznach, A., Zomet, O., Marom, A. & Yovel, Y. Conservation of brain connectivity and wiring across the mammalian class. *Nat. Neurosci.* **23**, 805–808 (2020).
8. Bryant, K. L., Li, L., Eichert, N. & Mars, R. B. A comprehensive atlas of white matter tracts in the chimpanzee. *PLoS Biol.* **18**, e3000971 (2020).
