# Supplementary material for: Detailed mapping of the complex fiber structure and white matter pathways of the chimpanzee brain
Source: Nat Methods. 2024 Jun 3;21(6):1122–30. doi: 10.1038/s41592-024-02270-1 (PMC11166572; doi:10.1038/s41592-024-02270-1)
Supplement: Supplementary file 2 — Reporting Summary [file 41592_2024_2270_MOESM2_ESM.pdf]

nature portfolio

Corresponding author(s): Dr. Cornelius Eichner

Last updated by author(s): Jan 16, 2024

Reporting Summary

Nature Portfolio wishes to improve the reproducibility of the work that we publish. This form provides structure for consistency and transparency in reporting. For further information on Nature Portfolio policies, see our Editorial Policies and the Editorial Policy Checklist.

Statistics

For all statistical analyses, confirm that the following items are present in the figure legend, table legend, main text, or Methods section.

n/a	Confirmed	
☒	☐	The exact sample size (n) for each experimental group/condition, given as a discrete number and unit of measurement
☒	☐	A statement on whether measurements were taken from distinct samples or whether the same sample was measured repeatedly
☒	☐	The statistical test(s) used AND whether they are one- or two-sided<br>*Only common tests should be described solely by name; describe more complex techniques in the Methods section.*
☒	☐	A description of all covariates tested
☒	☐	A description of any assumptions or corrections, such as tests of normality and adjustment for multiple comparisons
☒	☐	A full description of the statistical parameters including central tendency (e.g. means) or other basic estimates (e.g. regression coefficient) AND variation (e.g. standard deviation) or associated estimates of uncertainty (e.g. confidence intervals)
☒	☐	For null hypothesis testing, the test statistic (e.g. F, t, r) with confidence intervals, effect sizes, degrees of freedom and P value noted<br>*Give P values as exact values whenever suitable.*
☒	☐	For Bayesian analysis, information on the choice of priors and Markov chain Monte Carlo settings
☒	☐	For hierarchical and complex designs, identification of the appropriate level for tests and full reporting of outcomes
☒	☐	Estimates of effect sizes (e.g. Cohen's d, Pearson's r), indicating how they were calculated

Our web collection on statistics for biologists contains articles on many of the points above.

Software and code

Policy information about availability of computer code

Data collection	High Resolution Post-mortem MRI data were collected on a 9.4T Bruker Biospec 94/30 System, operated with Paravision version 6.01<br>Diffusion Pre-Scans were acquired on a 3T Siemens Connectom MRI System.
Data analysis	MRI data were processed and analzed using the following software:<br>- ANTs (v2.3.5)<br>- Braingl (v0.20170310)<br>- Bruker2NIfTI (v1.0.20180303)<br>- FSL (v6.0) and<br>- FSL eddy (build 508) for CUDA (v8.0)<br>- ITK-SNAP (v3.8.0)<br>- MRTRIX (v3.0.2)<br>- Python (v3.8) in conjunction with DiPy (v1.3.0), NiBabel (v3.1.1), NumPy (v1.18.5), SciPy (v1.5.0), and ScilPy (v1.1)<br><br>The data analysis pipeline is accessible under https://github.com/cornelius-eichner/EBC_dMRI_Preprocessing

For manuscripts utilizing custom algorithms or software that are central to the research but not yet described in published literature, software must be made available to editors and reviewers. We strongly encourage code deposition in a community repository (e.g. GitHub). See the Nature Portfolio guidelines for submitting code & software for further information.

Data

Policy information about availability of data

All manuscripts must include a data availability statement. This statement should provide the following information, where applicable:

- Accession codes, unique identifiers, or web links for publicly available datasets
- A description of any restrictions on data availability
- For clinical datasets or third party data, please ensure that the statement adheres to our policy

The resource presented here includes (i) dMRI data at 500 µm isotropic resolution, (ii) MR-microscopy FLASH data at 150 µm isotropic resolution, (iii) anatomical FLASH data at 500 µm isotropic resolution, (iv) WM fiber pathway reconstructions , (v) ultra-high-resolution TDI data, and (vi) segmentations of various anatomical brain structures. The raw data and all other features of the resource can be downloaded at https://openscience.cbs.mpdl.mpg.de/ebc/.
The data volume and 3D-reconstructed fascicles can be viewed online on the open science framework of the Max Planck Institute for Human Cognitive and Brain Sciences at https://openscience.cbs.mpdl.mpg.de/ebc/.

Code and processing routines are publicly available for download at https://github.com/cornelius-eichner/EBC_dMRI_Preprocessing.

Field-specific reporting

Please select the one below that is the best fit for your research. If you are not sure, read the appropriate sections before making your selection.

☒ Life sciences ☐ Behavioural & social sciences ☐ Ecological, evolutionary & environmental sciences

For a reference copy of the document with all sections, see nature.com/documents/nr-reporting-summary-flat.pdf

Life sciences study design

All studies must disclose on these points even when the disclosure is negative.

Sample size	One chimpanzee brain was selected for MRI data acquisition and measured twice with identical parameters.<br><br>One human in-vivo brain dataset was randomly selected from the 7T Human Connectome Project for visual comparison.<br>One human in-vivo brain dataset was randomly selected from the Human Connectome Project for Number of Fiber Orientations comparison.<br>One coronal human brain slab was obtained through the University of Leipzig's body donation program for MRI scanning and validation.<br><br>A sample size estimation was not applicable as this resource entails data from a single subject.
Data exclusions	No data were excluded.
Replication	To guarantee the stability of the results, an extensive test/retest analysis of the data set was performed.<br><br>The data collection was independently repeated once, two weeks after the initial collection. Data quality was confirmed to be reproducible in this experiment.
Randomization	Randomization was not required. The resource contains one brain sample.
Blinding	Blinding was not applicable in this study. Samples were analyzed identical and operator-independent procedures.

Reporting for specific materials, systems and methods

We require information from authors about some types of materials, experimental systems and methods used in many studies. Here, indicate whether each material, system or method listed is relevant to your study. If you are not sure if a list item applies to your research, read the appropriate section before selecting a response.

Materials & experimental systems

n/a	Involved in the study	
☐	☒	Antibodies
☒	☐	Eukaryotic cell lines
☒	☐	Palaeontology and archaeology
☐	☒	Animals and other organisms
☒	☐	Human research participants
☒	☐	Clinical data
☒	☐	Dual use research of concern

Methods

n/a	Involved in the study	
☒	☐	ChIP-seq
☒	☐	Flow cytometry
☐	☒	MRI-based neuroimaging

Antibodies

Antibodies used

Primary Antobodies:
1) Myelinated Fibers - Detected Protein: Myelin basic protein (MBP); Antibody: Rat anti-MBP; Dilution: 1:1000; Source: Abcam; Cat#: AB7349; Lot# GR3375915-1
2) Microglia - Detected Protein: Ionized calcium binding adaptor 1 (Iba-1); Antibody: Rabbit anti-Iba-1; Dilution: 1:2000; Source: Fujifilm; Cat#: Fujifilm 019-19741; Lot#: PTN5930
3) Astroglia - Detected Protein: Glial fibrillary acidic protein (GFAP); Antibody: Rabbit anti-GFAP; Dilution: 1:5000; Source: Dako; Cat#: Z0334; Lot#: 20059855
4) Aß plaques - Detected Protein: Amyloid-β 17–24; Antibody: Mouse anti-Aß clone 4G8; Dilution: 1:500; Source: Biolegend; Cat#: 800701; Lot#: B286227
5) Aß plaques - Detected Protein: Pyroglutamated amyloid-β Abeta-pE3; Antibody: Rabbit anti-pE3-Aß; Dilution: 1:500 Source: Synaptic Systems; Cat#: 218003; Lot#: 218003/6
6) Neurofibrillary Tangles - Detected Protein: hyperphosphorylated tau, pS202/pT205; Antibody: Mouse anti-pTau clone AT8; Dilution: 1:100; Source: Thermo Fisher Scientific; Cat#: MN1020

Secondary Antibodies:
Species: Donkey anti-Mouse; Labeling: biotinylated; Source: Dianova; Cat#: 715 065 150; Lot#: 144671
Species: Donkey anti-Rabbit; Labeling: biotinylated; Source: Dianova; Cat#: 711 065 152; Lot#: 147049
Species: Donkey anti-Rat; Labeling: biotinylated; Source: Dianova; Cat#: 712 065 150; Lot#: 124180

Validation

Primary antibodies 1 - 3 have been validated in the following study:
Morawski M., Kirilina E., Scherf N., Jäger C., Reimann K., Trampel R., Gavriilidis F., Geyer S., Biedermann B., Arendt T., Weiskopf N. Developing 3D microscopy with CLARITY on human brain tissue: Towards a tool for informing and validating MRI-based histology. NeuroImage 182: 417-428 (2018).

Primary antibodies 4 - 6 have been validated in the following study:
Schober R., Hilbrich I., Jäger C., Holzer M. Senile plaque calcification of the lamina circumvoluta medullaris in Alzheimer's disease. Neuropathology 41(5):366-370 (2021)

Animals and other organisms

Policy information about studies involving animals; ARRIVE guidelines recommended for reporting animal research

Laboratory animals

No laboratory animals were used in this study.

Wild animals

No wild animals were used in this study.

Data were acquired post-mortem from the brain of a deceased 47-year-old adult female chimpanzee (pan troglodytes verus) from Kolmården Wildlife Park, Sweden. The zoo chimpanzee was medically euthanized due to an untreatable cervical leiomyoma

Field-collected samples

No field collected samples were used in this study.

Ethics oversight

The procedures were in line with the ethical guidelines of primatological research at the Max Planck Institute for Evolutionary Anthropology, Leipzig, which were approved by the ethics committee of the Max Planck Society.

Note that full information on the approval of the study protocol must also be provided in the manuscript.

Magnetic resonance imaging

Experimental design

Design type

Structural Imaging

Design specifications

Not applicable, as no functional MRI data were collected.

Behavioral performance measures

Not applicable, as MRI data were post mortem.

Acquisition

Imaging type(s)

Diffusion and Structural MRI

Field strength

9.4T

Sequence & imaging parameters

Chimpanzee MRI data were acquired on a preclinical Bruker Biospec 94/30 MRI system at 9.4T (Paravision 6.0.1), using a 300 mT/m gradient system and a 154 mm transmit-receive quadrature RF coil (Bruker BioSpin, Ettlingen, Germany).

Diffusion MRI:

Segmented 3D EPI sequence with double adiabatic refocusing, TR = 1000 ms, TE = 58.9 ms, matrix size [r × p × s] = 240 × 192 × 144, no Partial Fourier, no parallel acceleration, EPI segmentation factor = 32 EPI-BW = 400 kHz. Diffusion-weighting was applied with b = 5.000 s/mm2 in 55 directions, uniformly distributed on a full sphere. Before the acquisition, ten diffusion-weighted volumes were acquired as dummy scans to achieve a constant steady-state temperature in the sample. Three interspersed b = 0 images without diffusion-weighting were acquired for field-drift correction. An additional b = 0 volume was acquired with reversed-phase encoding direction to correct off-resonance EPI distortions. A noise map with matching EPI parameters was additionally recorded to characterize the noise statistics of the dMRI data.

Anatomical Structural MRI:
Anatomical 3D FLASH MRI data were acquired using identical image dimensions and image resolution (500 µm isotropic) as the dMRI data: TR = 50 ms, TE = 9 ms, Matrix Size [r × p × s] = 240 × 192 × 144, no Partial Fourier, no parallel acceleration, BW = 20 kHz. To generate different contrasts, data with multiple flip angles were acquired with ? = [5, 12.5, 25, 50, 80]°. In addition, an ultra-high-resolution FLASH MR-microscopy dataset was acquired at 150 µm resolution using the following parameters: TR = 50 ms, TE = 9 ms, Matrix Size [r × p × s] = 800 × 640 × 480, ? = 27°, BW = 20 kHz.

The 500 µm dMRI and FLASH data acquisitions were repeated two weeks after the initial measurements for a test-retest evaluation.

Human MRI data were acquired on a preclinical Bruker Biospec 94/20 MRI system at 9.4T (Paravision 6.0.1), using a 660 mT/m gradient system and a 86 mm transmit-receive quadrature RF coil (Bruker BioSpin, Ettlingen, Germany).

Diffusion MRI:
Data with an isotropic resolution of 400 µm were acquired using a pulsed gradient diffusion weighted 2D Spin-Echo sequence using the following parameters: TR = 3074.5 ms, TE = 38.9 ms, matrix size [r × p × s] = 215 × 175 × 7, no Partial Fourier, no parallel acceleration, 9 averages. Diffusion-weighting was applied with b = 10.000 s/mm2 in 60 directions, on a half-sphere, and partially flipped to equally cover the full sphere. Each repetition contained 4 uniformly interspersed b=0 acquisitions without diffusion weighting.

Area of acquisition: whole brain (chimpanzee), coronal slab (human)

Diffusion MRI: ☒ Used ☐ Not used

Parameters: Chimpanzee: Diffusion MRI with 55 directions, single shell at b = 5.000 s/mm2. No cardiac gating
Human Diffusion MRI with 60 directions, single shell at b = 10.000 s/mm2. No cardiac gating

Preprocessing

Preprocessing software: Diffusion MRI preprocessing of the chimpanzee dataset entailed the following steps:
(i) Signal debiasing using Python (v3.8) in conjunction with autodmri (v0.2.5), DiPy (v1.3.0), NiBabel (v3.1.1), NumPy (v1.18.5), and SciPy (v1.5.0)
(ii) MP-PCA denoising using MRtrix (v3.0.2) dwidenoise
(iii) Volumetric Gibbs-ringing correction using MRtrix (v3.0.2) with custom built MRdegibbs3D
(iv) Field-drift correction using Python (v3.8) in conjunction with NiBabel (v3.1.1), NumPy (v1.18.5), and SciPy (v1.5.0)
(v) Eddy current and distortion correction using FSL (v6.0) eddy (build 508) for CUDA (v8.0)
(vi) DTI model fit using FSL (v6.0) dtifit
(vii) dMRI data normalization with non-diffusion-weighted volumes using Python (v3.8) in conjunction with NiBabel (v3.1.1), NumPy (v1.18.5)
(viii) Local Spherical Deconvolution using Python (v3.8) in conjunction DiPy (v1.3.0), NiBabel (v3.1.1), NumPy (v1.18.5), and SciPy (v1.5.0)
(ix) WM segmentaion using ITK-SNAP (v3.8.0)
(x) MR Tractography using MRTRIX (v3.0.2) tckgen

Normalization: The single subject data were not registered to a group template

Normalization template: N/A

Noise and artifact removal: Image noise was removed using MP PCA denoising, implemented in MRtrix (v3.0.2) dwidenoise

Volume censoring: N/A

Statistical modeling & inference

Model type and settings: N/A

Effect(s) tested: N/A

Specify type of analysis: ☒ Whole brain ☐ ROI-based ☐ Both

Statistic type for inference (See Eklund et al. 2016): N/A

Correction: N/A

Models & analysis

n/a	Involved in the study
☒	☐ Functional and/or effective connectivity
☒	☐ Graph analysis
☒	☐ Multivariate modeling or predictive analysis
